# Medication use, social determinants of health, and latent trajectories of functional outcomes in children with attention-deficit/hyperactivity disorder

**DOI:** 10.1371/journal.pone.0350337

**Published:** 2026-08-05

**Authors:** Margaret Fletcher, Wei Pan, Peter J. Duquette, Rachel Dew, Karin Reuter-Rice

**Affiliations:** 1 School of Nursing, Duke University, Durham, North Carolina, United States of America; 2 School of Nursing, University of North Carolina at Greensboro, Greensboro, North Carolina, United States of America; 3 Department of Population Health Sciences, School of Medicine, Duke University, Durham, North Carolina, United States of America; 4 Department of Psychiatry and Behavioral Sciences, School of Medicine, Duke University, Durham, North Carolina, United States of America; University of the West Indies at Saint Augustine, TRINIDAD AND TOBAGO

## Abstract

**Introduction:**

Children with attention-deficit/hyperactivity disorder (ADHD) are at increased risk for adverse long-term outcomes. However, little is known about how ADHD medication use and social determinants of health (SDOH) relate to children’s self-reported functional outcomes over time.

**Methods:**

This observational, longitudinal cohort study used data from the Adolescent Brain Cognitive Development (ABCD) Study®. Participants were nine to ten years old at enrollment, and analyses included the first five years of follow-up. Patterns of medication use were operationalized longitudinally. Latent class growth analysis was used to identify trajectory classes of child-reported family conflict, prosocial behavior, and school experiences. Multinomial logistic regression was used to examine associations between medication use patterns, SDOH, and functional outcome trajectory class membership.

**Results:**

Half of children used ADHD medication during the five-year period. Among medication users, the most common patterns included early initiation (medication use at study enrollment), stimulant-only use, and discontinuation without later reinitiation. Latent class growth analysis identified three trajectory classes across each functional domain: *Resilient*, *Declining*, and *Low Improving*. Social and structural factors were associated with trajectory membership, including ethnic discrimination, sex, insurance status, race and ethnicity, parental partnership and employment status, and medication type. Medication use patterns were not strongly associated with functional outcome trajectories; however, nonstimulant use (relative to stimulant-only use) was associated with less favorable outcomes. Females were more likely to demonstrate declining trajectories in family conflict and school experiences, whereas males were more likely to demonstrate low improving trajectories in prosocial behavior and school experiences. Ethnic discrimination was associated with less favorable trajectories across functional domains.

**Conclusions:**

Functional outcome trajectories in children with ADHD appear to be more strongly associated with social and structural factors than with patterns of medication use. These findings highlight the importance of addressing contextual influences when evaluating long-term functioning among children with ADHD.

## Introduction

In the United States (U.S.), approximately 11.3% of children have a parent-reported diagnosis of attention deficit hyperactivity disorder (ADHD) [1]. Medication treatment for ADHD has been widely studied, and robust evidence suggests it is safe and highly effective [2]. Despite the availability of effective treatment, children with ADHD face long-term challenges that negatively affect educational attainment [3–6], interpersonal relationships [6–11], and overall quality of life [12,13]. Stimulant medications are recommended as first-line treatment for individuals aged six years and older [14], although they can be associated with adverse effects including appetite suppression, sleep disturbance, and cardiovascular changes [2]. Nonstimulant medications, while less commonly used, have also demonstrated efficacy and may be prescribed alone or in combination with stimulants [2,15].

Early and consistent treatment of ADHD is hypothesized to improve long-term outcomes by reducing negative experiences at home, at school, and with peers during childhood [16–19]. Consistent with this hypothesis, numerous studies have reported beneficial effects of medication on functional and health outcomes across childhood, adolescence, and adulthood [20–23]. However, by comparing “medication- naïve” individuals against those with “any history” of stimulant use, current literature overlooks diverse patterns of exposure over time, including age at initiation and consistency of use. Furthermore, the long-term effects of nonstimulant medications remain poorly understood. Evidence from neuroimaging studies also suggests that stimulant effects are age-dependent, with early-onset treatment potentially yielding neurobiological advantages [24–27].

Although trajectories of ADHD symptom severity during childhood have been described, symptom severity alone does not capture the influence of evolving environmental demands on children’s daily functioning or the cumulative effects of ongoing ADHD-related challenges on children’s self-concept over time [16–19]. Functional outcome trajectories remain relatively understudied, despite ADHD diagnosis requiring evidence that symptoms interfere with functioning in at least two settings (e.g., at home, at school, or among peers) [28]. Prior research has identified several areas of functional difficulties among children with ADHD, including family conflict [7,29,30], stigmatization at school [29], impaired academic achievement [3,4,30], and impaired prosocial behavior [8,31,32]. Impaired prosocial behavior, in particular, has been associated with peer relationship difficulties and reduced quality of life [9]. Often, ratings of functional outcomes are provided by parents without taking into account the child’s perspective [33,34], and are not likely to reflect child functioning outside the home [30]. Additionally, children desire an increasing level of involvement in the management of their care as they progress into adolescence [35], and child-reported experiences provide valuable insight into the challenges they face [30,36–38].

Social determinants of health (SDOH) encompass the social, economic, and environmental conditions that influence health and functioning across the life course [39]. According to the U.S. Department of Health and Human Services Healthy People 2030 initiative and the National Academies of Sciences, Engineering, and Medicine, SDOH include five domains: economic stability; education access and quality; health care access and quality; neighborhood and built environment; and social and community context [39,40]. These domains are associated with inequities in ADHD prevalence, symptom burden, and access to treatment [1,15,41–49]. Importantly, the disparities linked to SDOH are not the result of individual characteristics but reflect systemic and structural inequities that influence access to resources. The extent to which these inequities influence functional outcomes in relation to patterns of ADHD medication use remains poorly understood.

Disparities related to sex further illustrate the influence of social context on ADHD diagnosis and treatment. Individuals assigned male at birth (hereafter referred to as “male”) are diagnosed with ADHD during childhood at approximately twice the rate of those assigned female at birth (hereafter referred to as “female”), a difference often attributed to more overt hyperactive symptom presentation among males and a predominance of inattentive symptoms among females, who may mask or compensate for their difficulties [50]. Despite lower rates of diagnosis, females with ADHD experience substantial academic, social, and mental health challenges, including anxiety, depression, and suicidal ideation [50–52]. Yet, females remain underrepresented in ADHD research and are less likely than males to be prescribed medication, even when symptom severity is comparable [53–55].

Racial and ethnic inequities in ADHD diagnosis and treatment are also well documented. Black children are reported to have lower prevalence rates of ADHD and are less likely than White children to receive a diagnosis prior to kindergarten [1,56,57], although these differences likely reflect underdiagnosis rather than true differences in prevalence [57]. A recent systematic review and meta-analysis conversely reported higher rates of ADHD among Black individuals, underscoring the need for improved detection and access to care [58]. Hispanic and Asian children similarly experience lower rates of diagnosis [1,57]. Following diagnosis, disparities persist in treatment utilization, as Black, Hispanic, and Asian children are significantly less likely than White children to fill prescriptions for ADHD medication [59]. Additionally, non-White adolescents are less likely to meet conventional thresholds for medication adherence, though such metrics do not provide nuanced understanding of the factors driving medication use patterns [52]. Experiences of racial and ethnic discrimination have also been identified by caregivers as barriers to care and family well-being [45].

Socioeconomic factors further intersect with these disparities. Children of parents with lower income or educational attainment are at increased risk for ADHD [47,60], and childhood ADHD is associated with reduced earnings and employment in adulthood [61]. Insurance status has also been linked to ADHD presentation and medication use, with children enrolled in Medicaid more likely to be identified as exhibiting hyperactive symptoms and more likely to receive combination therapy [15]. Neighborhood poverty has been associated with lower rates of medication use and greater symptom severity [42]. Additionally, parent partnership status (e.g., single- vs two-parent households) reflects variation in household social support and resources [62,63], and parent employment status represents economic stability [64,65]. Both family structure and employment have been linked to children’s mental/behavioral outcomes and to ADHD treatment patterns, including stimulant medication use [63,64]. The reasons for these inequities are unclear and could be related to biases of teachers and clinicians rather than characteristics of the children themselves. As such, these statistics should be considered in the context of complex sociocultural influences.

The present study aimed to address gaps in the literature by examining functional outcome trajectories among children with ADHD in relation to observed patterns of medication use within the broader context of SDOH, as detailed in the previously published study protocol [66]. Specifically, this study addressed the following aims:

1) Describe observed patterns of medication use over time, including age of initiation, types of medication used, and consistency of use.
2) Identify children with distinct latent trajectories of child-reported functional outcomes over time using latent class growth analysis.
3) Identify risk and resilience factors for functional outcome trajectories in children with ADHD using multinomial logistic regression.

By characterizing risk and resilience factors of functional outcome trajectories within complex social systems, this work intends to inform future interventions aimed at improving access to effective treatment and optimizing long-term outcomes for children with ADHD.

## Methods

### Design and data source

This is an observational, longitudinal cohort design, using data from the Adolescent Brain Cognitive Development (ABCD) Study® [67,68]. The ABCD Study® recruited 11,868 children aged 9–10 years old between 2016–2018 and will continue to follow them annually for 10 years, collecting neuroimaging, genetic/genomic, and survey data. Children were recruited from 21 sites across the U.S. As such, the large sample size and geographical coverage are a major strength of this database, and the 10-year longitudinal design provides opportunities for continued research throughout this developmental period. For the present study, de-identified ABCD Study® data release 6.0 was downloaded with permission from NIH Brain Development Cohorts (NBDC) Data Sharing Platform [69] on August 4, 2025. Data were available for time point 0 through time point 5 (T0-T5; 2016–2023). Authors did not have access to information that could identify individual participants at any point in time. The study was conducted and reported in accordance with the Strengthening the Reporting of Observational Studies in Epidemiology (STROBE) guidelines for observational research [70].

### Sample selection

The sample used for the present study was selected based on diagnostic criteria for current or past ADHD, which include the presence of at least six of nine inattentive and/or hyperactive symptoms and functional impairment across at least two settings prior to age 12 [28]. Participants who met diagnostic criteria for ADHD at the initial study visit were included in the sample, and children were excluded for history of diagnosed Bipolar 1, intellectual disability, psychosis, brain injury, cerebral palsy, epilepsy, lead poisoning, or alcohol use disorder as well as serious neurological conditions [71]. In total, **1,585** participants in the ABCD cohort met eligibility criteria and were included in the present study.

## Measures

All measures are described in Table 1. The computerized version of the Kiddie Schedule of Affective Disorders and Schizophrenia (KSADS-5) parent report was used for this study to assess for ADHD diagnostic criteria and symptom severity, bipolar I, anxiety, depression, oppositional defiant disorder, and conduct disorder [72]. The KSADS-5 was also used to assess inattentive and hyperactive symptom counts longitudinally [72]. Measures of family conflict, prosocial behavior, and school experiences were collected for each time point to measure functional outcomes. To assess family conflict, the ABCD Youth Family Environment Scale – Family Conflict Subscale was used (9 items; range: 0–1) [73–75], to assess prosocial behavior the Strengths and Difficulties Questionnaire – Prosocial Behavior Subscale was used (3 items; range: 0–6) [73,75,76] and to assess school experiences the School Risk and Protective Factors survey school environment (6 items; range: 6–24), school involvement (4 items; range: 4–16), and school disengagement (2 items; range: 2–8) subscales [77] were used. Factor scores were used to create a standardized, appropriately weighted composite measure reflecting school experiences broadly. The compositive school experiences score yielded acceptable internal consistency (Table 1). To facilitate interpretability and comparability across outcomes, family conflict and prosocial behavior means were used to create standardized z-scores for each of these measures. Additionally, the ABCD Annual COVID Survey – Parent and the COVID-19 Questionnaire – Parent from the ABCD COVID-19 sub study were used to derive a measure of COVID-19-related school disruptions, as this could theoretically have a significant confounding effect on functional outcomes, particularly in children with ADHD [78–83].

**Table 1:** Measures [66].

Variables	Measure and Source	Full Cohort^1^	ADHD Sample^1^
Medication use (type, timing, and consistency of use)	ABCD Parent Medications Survey Inventory^2^ **(Parent)**	N/A	N/A
Symptom counts	KSADS-5 – Parent Diagnostic Interview **(Parent)**	N/A	N/A
ADHD diagnosis			
Internalizing symptoms	Child Behavior Checklist (CBCL) Internalizing and Externalizing Subscales **(Parent)**	.73-.79	.72-.79
Externalizing symptoms		.84-.85	.83-.86
Sex	ABCD Demographics Survey^2^ **(Parent)**	N/A	N/A
Race and ethnicity			
Household income (SDOH-*Economic stability)*			
Highest parental education (SDOH-*Education access and quality)*			
Insurance (SDOH-*Healthcare access and quality)*			
Parental partnership and employment (SDOH-*Economic stability, social and community context)*			
Neighborhood deprivation (SDOH-*Neighborhood and built environment)*	Area of Deprivation Index (ADI) **(Linked external data)**	N/A	N/A
Perceived discrimination (SDOH-*Social and community context)*	Perceived Discrimination Scale (T2 only) **(Youth)**	.76	.88
Family conflict	ABCD Youth Family Environment Scale – Family Conflict Subscale^2^ **(Youth)**	.65-.72	.65-.72
Prosocial behavior	Strengths and Difficulties Questionnaire – Prosocial Behavior Subscale **(Youth)**	.53-.68	.56-.71
School experiences composite scale	School Risk and Protective Factors^2^ **(Youth)**	.72-.81	.68-.75

^1^Internal consistency (Cronbach’s alpha)

^2^Adapted from Phenotypes and Exposures (PhenX) toolkit [85]

Demographics and SDOH variables used in this study were obtained from the ABCD Parent Demographics Survey and include sex (male and female), and racial and ethnic identity (White, Black, Hispanic, Another racial or ethnic identity), annual household income, highest level of parent education, parent partnership and employment status, and insurance status. Additionally, the Area Deprivation Index (ADI) was used to represent the neighborhood and built environment, and the Perceived Discrimination Scale was used to measure experiences of racial or ethnic discrimination (7 items; range: 1–5). Covariates, including standardized scores for internalizing (i.e., depression and anxiety) and/or externalizing (i.e., oppositional defiant and conduct) disorders at study enrollment, were assessed using the Child Behavior Checklist (CBCL) [84]. Insurance status and perceived discrimination were not assessed at study enrollment, thus the earliest available data for these variables (T2) were used. Detailed descriptions and psychometric properties for all measures, including derivation methods for the COVID-19-related school disruptions covariate are available in the previously published protocol [66].

To assess medication use, the ABCD Parent Medications Survey Inventory, adapted from the Phenotypes and Exposures (PhenX) toolkit was used [85]. With this questionnaire, parents provided a two-week history of the name, frequency, and dosage of any medications taken by the child at each time point. Medications were coded as either stimulant (e.g., methylphenidate and amphetamine medications) or nonstimulant (e.g., atomoxetine and alpha-2 agonists) based on names of FDA-approved medications for the treatment of ADHD [86]. Medication use was described per time point as “none”, “stimulant only”, “nonstimulant only”, or “both”. Variables were then created based on patterns of use across time. Participants taking medication at the time of study enrollment were categorized as “early” age of initiation, while participants who first reported medication use at later time points (T1-T5) were categorized as “late”. Participants were categorized as either using only stimulant medication throughout the duration of the study or as taking a nonstimulant at any time point, either with or without a stimulant. Consistency was defined by medication use at the time points following initiation, with “consistent” use defined as taking medication at every time point following initiation, “inconsistent” use defined as stopping and subsequently restarting medication, and “discontinued” use was defined as stopping medication without resuming. Participants who reported no medication use across all time points were categorized as “never used” (Table 2). Medication use in the ABCD Study® cohort has been previously examined at time points T0-T3 and was operationalized similarly, with the baseline study time point representing a cut-off for early use, and initiation at any subsequent time point representing later use [87].

**Table 2:** Operationalization of medication use patterns.

Medication Pattern	Levels	Definition
Medication type	Stimulant only	Only stimulant use across all time points
	Some nonstimulant	Some nonstimulant use at any time point, with or without stimulant use
Age of initiation	Early	Taking medication at the initial study time point (T0)
	Late	Initial medication time point between T1-T5
Consistency	Consistent	Continued taking medication at all time points following initiation
	Inconsistent	Discontinued and later re-initiated medication use
	Discontinued	Discontinued and did not re-initiate

T0: time point 0 (initial study visit); T1-T5: time point 1–5 (annual follow-up study visits)

### Analytic strategy

#### Missing data.

Missing data were addressed using a combination of maximum likelihood estimation and imputation strategies. For Aim 2, data were assumed missing at random (MAR) and models were estimated using maximum likelihood methods, which retain participants with partially observed longitudinal data. For Aims 1 and 3, time-fixed variables with missingness <5% were imputed using mean imputation for continuous and mode imputation for categorical variables. When missingness exceeded 5%, Little’s MCAR test was used and expectation-maximization (EM) imputation was applied when data were not missing completely at random [88].

For time-varying predictors and covariates in Aim 3, multilevel models were used to evaluate change over time and determine whether predictors should be treated longitudinally (intercept/slope/quadratic terms) or represented by a single time point. In the former case, missing data were handled by maximum likelihood estimation in multilevel models. In the latter case, missing values in the representative single time point (T0 when available; T2 for insurance status and ethnic discrimination measures) were imputed using observed responses from other time points when possible. Additional details on missing data procedures are provided in the published protocol and accompanying supplementary materials [66].

### Aim 1

Medication use patterns were summarized using descriptive statistics. Frequencies and proportions were calculated to describe medication type (i.e., stimulant, nonstimulant), age of initiation (i.e., early, late), and consistency of use (i.e., consistent, inconsistent, discontinued) across study time points. These descriptive analyses were used to characterize observed longitudinal patterns of medication use, allowing for use as a time-fixed predictor in subsequent analyses.

### Aim 2

Latent class growth analysis (LCGA) was used to identify distinct latent classes of functional outcome trajectories over the first six time points of the ABCD Study®. We chose Latent Class Growth Analysis (LCGA) over Growth Mixture Modeling (GMM) because LCGA assumes homogenous growth trajectories within each class, emphasizing between-class contrasts and results in a more parsimonious model that is less prone to convergence issues. As this study aimed to evaluate between-class differences rather than person-specific random effects within classes, we believe this is the most appropriate and clinically interpretable method [89–92]. Time was modeled as study time point (T0-T5), including linear and quadratic trends. Correlations between outcomes were small (Pearson’s *r* = 0.21–0.33) and thus, family, prosocial, and school outcomes were modeled separately rather than creating a composite score as was originally intended [66].

The COVID-19 pandemic occurred during this study time frame and resulted in widespread disruption of schooling, which has been shown to have adverse effects on functioning and mental health in children and adolescents, and those with ADHD are particularly vulnerable to these effects [78–83]. As such, data collected by the ABCD Study® and ABCD COVID-19 sub-study regarding COVID-19-related school disruptions were used to derive a COVID-19-related school disruptions variable. This variable was then included as a time-varying covariate in the latent class models. We determined the best-fitting trajectory model using several fit indices, including the Akaike Information Criterion (AIC), the Bayesian Information Criterion (BIC), and entropy. We prioritized the BIC because it is a more conservative indicator that penalizes model complexity, helping to prevent overfitting. Additionally, entropy, model parsimony, and interpretability were considered when selecting the final class solutions [92]. Lastly, sample weights were not included in this model, as they were accounted for in Aim 3.

### Aim 3

The relationships between independent variables (sex, race, ethnicity, income, parental education, insurance status, ADI, ethnic discrimination, parental partnership and employment, and medication use patterns) and the dependent variables (functional outcome trajectory class membership) were evaluated using multinomial logistic regression. ABCD Study® sample weights were included in the model to support the generalizability of the results. Baseline comorbid internalizing and externalizing symptoms (CBCL t-scores) were included as time-fixed covariates, and inattentive and hyperactive symptom counts were included as time-varying covariates in the model. Independent variables were assessed for multicollinearity using correlation matrices and variance inflation factors (VIFs), and maximum likelihood estimation was used. Model fit was evaluated using likelihood ratio tests, pseudo-R^2^, and area under the receiver operating characteristic (AUROC). Odds ratios and 95% confidence intervals were reported for each individual variable. G*Power was used to conduct *a priori* power analysis, which suggested adequate power to detect a small estimated effect size (OR = 1.2). Significance was reported at *p* = .05 and *p* = .017 following Bonferroni correction for multiple comparisons.

## Results

A total of 1,587 participants met eligibility criteria at baseline. Two participants were later excluded from trajectory analyses because all three functional outcome measures were missing at all time points, resulting in a final analytic sample of **N = 1,585**. Across study waves, the sample size decreased to 1109 at T5, representing 30.1% attrition from baseline. Baseline demographic characteristics of participants retained compared to those lost to follow-up are included in S1 Table. Missingness was handled using maximum likelihood estimation in trajectory models under a MAR assumption.

The sample was majority male (67%), White (53.7%), with high levels of parent education (56.6% with a bachelor’s degree or higher), and high income (34.5% making $100,000 or more annually). Participants were aged 9–10 at baseline and had private insurance (56.1%) and their parents were most often partnered, with one or both partners active in the labor force (65.9%). Approximately half of the sample met diagnostic criteria for past-only ADHD, and combined (i.e., inattentive and hyperactive) was the most common presentation at baseline. Baseline sample characteristics are further detailed in Table 3.

**Table 3:** Baseline sample characteristics (N = 1,585).

Variable name	n (%)	Variable name	n (%)
**Sex**		**Parent partnership and employment status**	
Male	1063 (67.0)	Single, not in the labor force	161 (10.1)
Female	524 (33.0)	Single, in the labor force	313 (19.7)
**Racial and ethnic identity**		Partnered, 0 partners in the labor force	43 (2.7)
Hispanic	291 (18.3)	Partnered, 1 partner in the labor force	352 (22.2)
White	853 (53.7)	Partnered, 2 partners in the labor force	693 (43.7)
Black	237 (14.9)	**ADHD criteria met**	
Another racial or ethnic identity	206 (13.0)	Past–only	697 (43.9)
**Highest education across parents**		Current ADHD	765 (48.3)
Up to high school	69 (4.3)	Partial remission	125 (7.8)
High school diploma/GED	130 (8.2)	**ADHD baseline presentation**	
Some college	491 (30.9)	Combined	362 (22.8)
Bachelor’s degree	425 (26.8)	Inattentive	319 (20.1)
Graduate or professional degree	470 (29.6)	Hyperactive	86 (5.4)
**Income**		Remitted or partially remitted	820 (51.7)
<25k	246 (15.5)	**Variable name**	**Mean (SD)**
25k–50k	237 (14.9)	**ADI**	41.79 (27.45)
50k–75k	211 (13.3)	**Ethnic discrimination** ^1^	1.22 (.42)
75k–100k	219 (13.8)	**Internalizing symptoms (t–score)**	55.65 (10.7)
100k–200k	418 (26.3)	**Externalizing symptoms (t–score)**	55.4 (10.5)
>200k	130 (8.2)	**ADHD symptom counts**	
Don’t know	66 (4.2)	Inattentive	3.8 (3.7)
Prefer not to answer	60 (3.8)	Hyperactive	3.0 (3.3)
**Insurance status** ^1^		**Functional outcomes**	
Private	807 (56.1)	Family conflict (range = 0–1)	.27 (.23)
Public	452 (31.4)	Prosocial behavior (range = 0–2)	1.6 (.4)
Uninsured	49 (3.4)	School Involvement (range = 1–4)	3.1 (.7)
Multi–insurance	105 (7.3)	School Environment (range = 1–4)	3.2 (.5)
Other insurance	18 (1.2)	School Disengagement (range = 1–4)	2.0 (.8)

^1^Not measured at baseline, values reflect earliest available measurement (T2)

### Aim 1: Patterns of medication use over time

Medication use patterns in the sample are described in Table 4. Three distinct, observable aspects of medication use were identified: medication type, age at initiation, and consistency of use over time. Half of participants (50.2%) did not use medication at any point during the duration of the study, while 30.6% only used stimulant medication and 19.2% used a nonstimulant medication at some point during the study period. Early medication initiation was defined as using medication at the time of study enrollment (32.7%), while late initiation included all participants who were not using medication at study enrollment but initiated medication use at any time point thereafter (17.1%). Consistency was defined as either “consistent” (i.e., starting medication and continuing at all subsequent study time points; 12.9%), “inconsistent” (i.e., starting, stopping, and re-starting medication across study time points; 11.9%), or “discontinued” (i.e., stopping medication without resuming at a future time point; 25.6%). Overall medication use decreased over time, with the proportion of participants reporting no medication use increasing from 67.3% at T0 to 71.8% at T5.

**Table 4:** Patterns of medication use.

Time point (T0-T5)						
	**T0**	**T1**	**T2**	**T3**	**T4**	**T5**
**None**	1067 (67.3%)	998 (66.9%)	944 (65.6%)	883 (67.4%)	852 (68.9%)	796 (71.8%)
**Stimulant only**	366 (23.1%)	342 (22.9%)	341 (23.7%)	297 (22.7%)	274 (22.1%)	229 (20.7%)
**Nonstimulant only**	63 (4.0%)	69 (4.6%)	70 (4.9%)	57 (4.4%)	45 (3.6%)	32 (2.9%)
**Both**	89 (5.6%)	82 (5.5%)	83 (5.8%)	72 (5.5%)	66 (5.3%)	52 (4.7%)
**Observable medication use patterns over time**						
		**Never used** 796 (50.2%)				
**Medication type**		**Age of initiation**			**Consistency of use**	
**Stimulant only**	485 (30.6%)	**Early**	518 (32.7%)	**Consistent use**	196 (12.9%)	
**Some nonstimulant**	304 (19.2%)	**Late**	271 (17.1%)	**Inconsistent use**	188 (11.9%)	
				**Discontinued**	405 (25.6%)	

**Fig 1:**
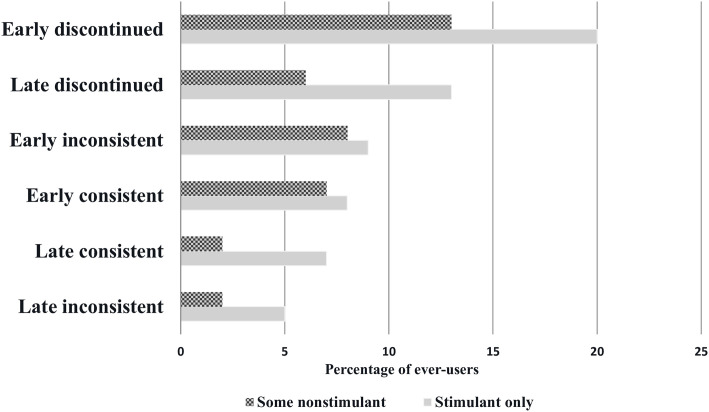
Relationships among patterns of medication use.

Bivariate comparisons demonstrated that early users were more likely to use nonstimulant medication at some point (*p* < .001), and while early users were more often inconsistent; late users were more often consistent (*p* = .048). Fig 1 illustrates the heterogenous patterns of medication use. Early and discontinued was the most prevalent pattern of medication timing and consistency, while late consistent and late inconsistent were the least prevalent.

Chi-Square bivariate associations between medication use and demographic variables were tested and significant associations were found between medication use patterns and sex, racial and ethnic identity, income, insurance status, parent education, and parent partnership and employment (S2 Table). Female participants were less likely to ever use medication, to use it early, or to be prescribed a nonstimulant. White and higher income participants were more likely to ever use medication, to use it early, and to use medication consistently compared to minoritized racial and ethnic identities and lower income groups. Higher parent education was associated with higher likelihood of ever using medication and greater consistency of use, but lower parent education levels were associated with higher likelihood of being prescribed a nonstimulant. Private insurance was associated with more consistent use and lower likelihood of nonstimulant prescription. Having partnered parents with both partners in the workforce was significantly associated with higher likelihood of medication use, earlier use, and more consistent use.

### Aim 2: Functional outcome latent trajectory classes

Correlations between family conflict, prosocial behavior, and school outcomes were small (*r* = 0.21–0.33), supporting the use of separate latent class growth analysis (LCGA) models for each functional outcome trajectory. Models included fixed effects for linear and quadratic time (time and time²) and a covariate capturing COVID-19–related school disruptions. Within-class variability was constrained by fixing random effects to zero, allowing class differences to reflect distinct patterns of change over time. Across all outcomes, a three-class solution demonstrated superior fit, including lower BIC, acceptable entropy, and interpretability (S3 Table). Although the four-class solution for school experiences demonstrated slightly improved fit indices compared to the three-class solution, the additional class was not meaningfully distinct from the other classes and did not yield substantively different associations with predictors or covariates in bivariate analyses. Therefore, the three-class solution was retained due to its greater interpretability. Mean posterior probabilities ranged from 0.71 to 0.89, indicating adequate classification precision across classes [93].

**Fig 2:**
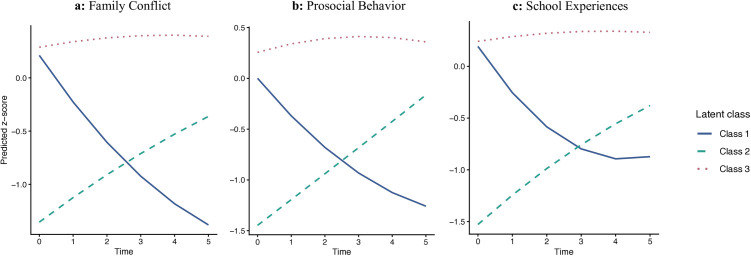
Predicted Functional Outcome Trajectories by Latent Class.

A three-class solution revealed similar trajectory patterns across all three functional outcomes. Specifically, classes differed significantly in baseline levels and longitudinal change, with *Declining* (Class 1) characterized by declining trajectories (family: n = 204 [12.9%]; prosocial: n = 260 [16.4%]; school: n = 230 [14.5%]), *Low Improving* (Class 2) showing an initial low baseline followed by partial improvement (family: n = 245 [15.5%]; prosocial: n = 277 [17.5%]; school: n = 183 [11.5%]), and *Resilient* (Class 3) reflecting stable high/resilient functioning over time (family: n = 1,136 [71.7%]; prosocial: n = 1,147 [72.4%]; school: n = 1,172 [73.9%]) (Fig 2). Demographic characteristics of the three latent classes for each outcome are detailed in S4 Table.

### Aim 3: Predictors of functional outcome latent class membership

Multinomial logistic regression models were run separately for family conflict, prosocial behavior, and school experiences trajectory classes. Predictors and covariates demonstrated minimal collinearity, with variance inflation factors (VIFs) <4 for all terms. Expected correlations between inattentive and hyperactive symptom parameters were observed, and some dependencies across dummy-coded medication variables (e.g., never used categories) were present, but model estimation remained stable. Sample weights provided by the ABCD Study® were included in all models. Because the *Resilient* class was the largest and reflected the most favorable trajectories across outcomes, it was selected as the reference category. Therefore, adjusted odds ratios (aORs) reflect likelihood of membership in the *Declining* or *Low Improving* classes relative to the *Resilient* class. ADHD diagnostic status (past-only vs current/partially remitted) was examined in preliminary bivariate analyses and was not significantly associated with functional outcomes; as such, it was not included in final adjusted models. Sensitivity analyses including this variable also did not reveal any meaningful significant associations. In medication-only models, nonstimulant exposure was associated with increased odds of membership in the *Declining* family class and both the *Declining* and *Low Improving* prosocial classes. Across models, ethnic discrimination consistently predicted membership in non-resilient classes, while nonstimulant exposure was most strongly associated with declining family/prosocial trajectories, and public or multiple (i.e., both public and private) insurance status was most strongly associated with declining school trajectories. Multinomial logistic regression results, including adjusted odds ratios and confidence intervals for all predictors and covariates are reported in S5 Table, while significant findings are described hereafter.

### Declining vs. Resilient

#### Family conflict.

Membership in the *Declining* family conflict class was characterized by initially positive child-reported family conflict experiences (i.e., lower levels of family conflict), followed by marked decline across time with a significant positive quadratic curve, with children reporting more negative family conflict experiences by T5. Higher experiences of ethnic discrimination (*aOR* = 1.93, 95% CI [1.29, 2.88], *p* = .001), history of some nonstimulant medication use compared to stimulant-only use (*aOR* = 1.77, 95% CI [1.11, 2.81], *p* = .016), and female sex (*aOR* = 1.50, 95% CI [1.08, 2.07], *p* = .016) were associated with increased odds of *Declining* family conflict class membership. Pairwise contrasts did not reveal any significant differences by racial or ethnic identity.

#### Prosocial behavior.

Membership in the *Declining* prosocial behavior class was characterized by initially average child-reported prosocial behavior followed by declining levels across time with a significant positive quadratic curve. Public insurance was associated with increased odds of *Declining* prosocial behavior class membership compared to private insurance (*aOR* = 1.57, 95% CI [1.07–2.31], *p* = .022), as was some nonstimulant medication use when compared to stimulant-only use (*aOR* = 1.72, 95% CI [1.15–2.57], *p* = .008). Having partnered parents with neither partner in the workforce was associated with decreased odds of *Declining* prosocial behavior class membership compared to families with two working parents (*aOR* = 0.33, 95% CI [0.13, 0.85], *p* = .021), and female sex was also associated with decreased odds of *Declining* prosocial behavior class membership (*aOR* = 0.58, 95% CI [0.42, 0.79], *p* = .001). Pairwise contrasts did not reveal any significant differences by racial or ethnic identity.

#### School experiences.

Membership in the *Declining* school experiences class was characterized by initially positive child-reported school experiences followed by a modest decline by T5. Public insurance (*aOR* = 2.44, 95% CI [1.64, 3.62], *p* < .001) and multiple types of insurance (*aOR* = 2.69, 95% CI [1.60, 4.51], *p* < .001) were both associated with higher odds of membership in the *Declining* school experiences class when compared to private insurance, and female sex was also associated with increased odds of *Declining* school experiences class membership (*aOR* = 1.36, 95% CI [1.01–1.83], *p* = .042). In contrast, Black race (*aOR* = 0.45, 95% CI [0.27, 0.74], *p* = .002) and participants with Another racial or ethnic identity (*aOR* = 0.50, 95% CI [0.28, 0.91], *p* = .023) were associated with lower odds of *Declining* school experiences class membership relative to White participants. Additional pairwise contrasts revealed that Black race (*aOR* = 0.42, 95% CI [0.25, 0.71], *p* = .001) and Another racial or ethnic identity (*aOR* = 0.48, 95% CI [0.26, 0.89], *p* = .019) were also associated with lower odds of membership in the *Declining* school experiences class compared to Hispanic ethnicity.

### Low Improving vs. Resilient

#### Family conflict.

Higher neighborhood deprivation (ADI) (*aOR* = 1.01, 95% CI [1.00–1.02], *p* = .004) and higher experiences of ethnic discrimination (*aOR* = 1.85, 95% CI [1.27–2.70], *p* = .001) were associated with increased odds of *Low Improving* family conflict class membership, while Hispanic ethnicity was associated with decreased odds of membership in the *Low Improving* family conflict class relative to White participants (*aOR* = 0.55, 95% CI [0.36–0.84], *p* = .006). Pairwise contrasts did not reveal any other significant differences by racial or ethnic identity.

#### Prosocial behavior.

Membership in the *Low Improving* prosocial behavior class was characterized by initially negative child-reported prosocial behavior followed by improvement over time, though outcomes remained less positive than the *Resilient* class. Higher experiences of ethnic discrimination were associated with increased odds of *Low Improving* prosocial behavior class membership (*aOR* = 1.60, 95% CI [1.03, 2.49], *p* = .036). Black race was associated with decreased odds of *Low Improving* prosocial behavior class membership relative to White participants (*aOR* = 0.38, 95% CI [0.19–0.76], *p* = .007), and female sex was also associated with decreased odds of membership in the *Low Improving* prosocial behavior class (*aOR* = 0.47, 95% CI [0.32, 0.70], *p* < .001). Pairwise contrasts revealed that Black participants also differed significantly from Hispanic participants (*aOR* = 0.31, 95% CI [0.15–0.62], *p* = .001).

#### School experiences.

Membership in the *Low Improving* school experiences class was characterized by initially negative child-reported school experiences followed by improvement over time, though outcomes remained less positive than the *Resilient* class. Higher experiences of ethnic discrimination were associated with increased odds of *Low Improving* school experiences class membership (*aOR* = 1.54, 95% CI [1.00, 2.37], *p* = .048), and female sex was associated with decreased odds of *Low improving* school experiences class membership (*aOR* = 0.65, 95% CI [0.45, 0.95], *p* = .025). Pairwise comparisons also revealed that Another racial or ethnic identity was associated with lower odds of *Low Improving* school experiences class membership compared to Hispanic ethnicity (*aOR* = 0.52, 95% CI [0.28, 0.97], *p* = .040).

#### Covariates.

**Fig 3:**
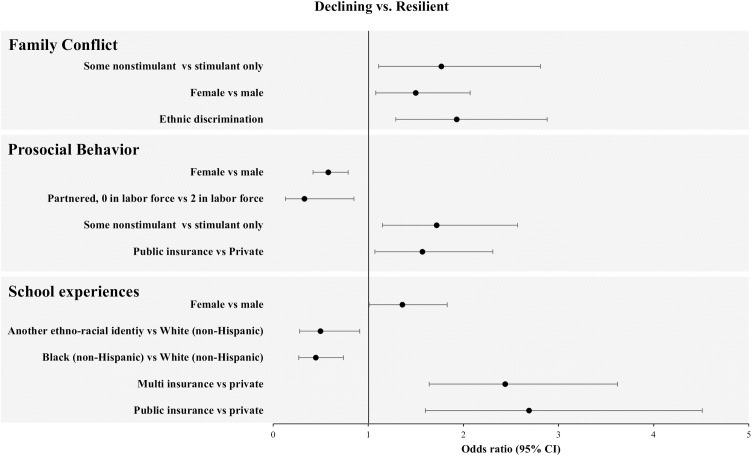
Forest plot for *Declining* versus *Resilient* Trajectories.

**Fig 4:**
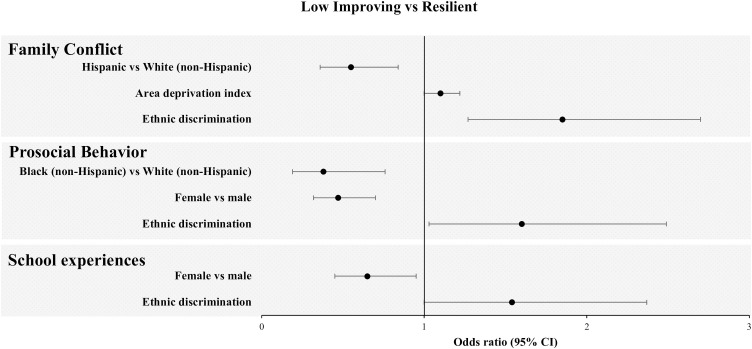
Forest plot for *Low Improving* versus *Resilient* Trajectories.

Models adjusted for baseline internalizing and externalizing symptoms and ADHD symptom trajectories (inattentive and hyperactive symptom intercepts, slopes, and quadratic terms). After adjustment, higher baseline externalizing symptoms were consistently associated with greater odds of membership in less favorable trajectory classes, including the *Declining* (*aOR* = 1.03, 95% CI [1.01, 1.05], *p* = .004) and *Low Improving* (*aOR* = 1.04, 95% CI [1.03, 1.06], *p* < .001) classes for family conflict, and the *Declining* (*aOR* = 1.02, 95% CI [1.00, 1.04], *p* = .016) and *Low Improving* (*aOR* = 1.02, 95% CI [1.00, 1.05], *p* = .021) classes for prosocial behavior. Baseline internalizing symptoms were generally not associated with class membership, with the exception of school experiences, where higher internalizing symptoms were associated with increased odds of membership in the *Low Improving* school class (*aOR* = 1.04, 95% CI [1.02, 1.06], *p* < .001). ADHD inattentive and hyperactive symptom growth parameters also demonstrated independent associations with trajectory class membership across outcomes, indicating that relationships of predictor variables with functional outcome trajectories were robust to differences in baseline symptom burden and longitudinal symptom change. Adjusted odds ratios (*aOR*s) for all significant predictors are detailed in Figs 3 and 4.

After full adjustment, ethnic discrimination was the most consistent predictor of non-resilient trajectories, showing associations across outcome domains. Several predictors also showed domain-specific patterns. For family conflict, nonstimulant exposure and female sex were associated with the *Declining* trajectory, while higher neighborhood deprivation (ADI) and baseline externalizing symptoms were associated with the *Low Improving* trajectory, and Hispanic identity was associated with lower odds of *Low Improving* membership. For prosocial behavior, nonstimulant exposure, public insurance, and baseline externalizing symptoms were associated with the *Declining* trajectory, while female sex and Black identity were associated with lower odds of *Low Improving* membership. For school experiences, insurance status showed the strongest associations, with public and multiple insurance policies linked to the *Declining* trajectory, and baseline internalizing symptoms associated with the *Low Improving* trajectory. In contrast, age of medication initiation and consistency of use were not significantly associated with trajectory class membership in adjusted models. Risk and resilience factors for class membership are detailed in Table 5.

**Table 5:** Risk and resilience factors for functional outcome trajectory class membership.

	Risk Factors^1^		Resilience Factors^2^
	Class 1: *Declining*	Class 2: *Low Improving*	Class 3: *Resilient*
**Family conflict**	Nonstimulant use	Neighborhood deprivation	Stimulant only (declining)
	Female	Ethnic discrimination	Hispanic identity (low improving)
	Ethnic discrimination		
**Prosocial behavior**	Nonstimulant use	Ethnic discrimination	Partnered parents, neither working (declining)
	Male	Male	Black (non-Hispanic) (low improving)
	Public insurance		
**School experiences**	Public or multiple insurance	Ethnic discrimination	Black (non-Hispanic) (declining)
		Male	Another identity (declining)

^1^Comparisons with *Resilient* class membership as reference group

^2^Resilience factor in comparison to latent class noted in parentheses

## Discussion

Children with ADHD demonstrated heterogeneous patterns of medication use and functional outcome trajectories throughout early adolescence, and few associations were found between medication use and child-reported functional outcomes. Medication discontinuation was common, consistent with prior research showing that long-term persistence of ADHD medication use is often low in real-world settings [94]. Additionally, bivariate associations echo previously described disparities in medication access [87,95–97]. For family conflict and prosocial behavior, nonstimulant medication use was associated with greater odds of membership in the *Declining* trajectory class. This pattern does not represent a causal relationship of nonstimulant use on less desirable outcomes but rather is likely an indicator of greater clinical complexity among children who receive nonstimulants. Prior evidence suggests nonstimulants are particularly useful in the context of children with more prominent externalizing symptoms or an inadequate response to stimulant monotherapy [98]. In contrast, age of initiation and consistency of medication use were not predictive of functional outcome class membership, suggesting that medication timing and continuity were not robust correlates of child-reported functional outcome trajectories in this cohort. One possible explanation for these findings is that school services, parent training, and other non-pharmacological therapies were not captured in these data, which are important components of ADHD management [99]. Additionally, prior longitudinal cohort research has likewise found that early symptom-course patterns demonstrate greater prognostic value than medication or other treatment [100]. These findings should not be interpreted as evidence that medication is ineffective but instead underscore the inherent complexity of evaluating treatment effects in naturalistic settings, where treatment use and outcomes reflect numerous clinical and contextual factors.

Ethnic discrimination, however, emerged as a consistent risk factor across outcomes. Ethnic discrimination was associated with both the *Declining* and *Low Improving* family conflict trajectories, and with *Low Improving* trajectories for prosocial behavior and school experiences. These findings align with prior work establishing the reliability, validity, and predictive utility of the ABCD Study® Perceived Discrimination Scale, including evidence that higher discrimination is associated with unfavorable outcome trajectories, including increases in internalizing and somatic symptoms over time [101]. Ethnic discrimination has also been linked to psychotic-like experiences in the ABCD cohort [102], emphasizing the broader mental health relevance of discrimination experiences during adolescence. Additionally, ethnic discrimination has been associated with poorer academic functioning, increased risk behaviors, and physical health manifestations, contributing to heightened allostatic load during this developmental stage, particularly in minoritized racial and ethnic groups [103,104]. Importantly, prior research suggests that the effects of ethnic discrimination may be ameliorated by protective factors such as parental warmth and supportive racial and ethnic socialization [103,105–109]. Supporting parents in providing these measures and advocating for school environments that promote diversity and equitable treatment of students of all backgrounds are plausible foundations for intervention to reduce or buffer against experiences of ethnic discrimination [103,110].

Adjusted for ethnic discrimination, racial and ethnic identity remained significantly associated with trajectory class membership as well. Hispanic children had significantly lower likelihood of membership in the *Low Improving* class for family conflict compared to White children, and Black children were significantly less likely to fall into the *Low Improving* class for prosocial behavior or the *Declining* class for school experiences. These findings may reflect the presence of unmeasured protective processes that support positive adaptation in the context of structural inequities. While Hispanic identity reflects a broad and highly heterogeneous population, encompassing diverse cultural backgrounds and immigration histories, these findings should be interpreted with caution and should not be assumed to represent a uniform experience across all Hispanic children and families. Nonetheless, one plausible explanation for more favorable trajectories among some Hispanic participants may include protective mechanisms, such as family cohesion and strong familial support [111]. Similarly, participants categorized as Another racial or ethnic identity were also less likely to be classified in the *Declining* school experiences trajectory; however, this group is also heterogeneous and likely includes youth with diverse backgrounds, warranting cautious interpretation. These results highlight the importance of considering both resilience-promoting processes and structural context when interpreting associations of racial and ethnic identity and functional outcome trajectories in children with ADHD.

Public insurance and multiple insurance coverage were associated with greater odds of membership in the *Declining* school trajectory class. This pattern likely reflects insurance type as an indicator of broader structural context, rather than a direct causal effect of coverage itself. Medicaid is also a key source of support for school-based health and behavioral services, and greater reliance on publicly funded coverage may indicate more complex health and learning needs that place students at risk for worsening school experiences over time [112]. Although policy-level evidence suggests that expanding access to public coverage can improve child education outcomes on average [113], in this clinical cohort, public health insurance enrollment may represent higher baseline needs and structural risk.

Lastly, sex-based differences in outcome trajectories were also apparent. Despite being less likely to receive medication in bivariate analyses, females demonstrated more favorable prosocial behavior trajectories, consistent with prior literature describing sex differences in prosocial functioning [114]. However, females had higher odds of membership in the *Declining* class for family conflict and school experiences. Females were also less likely to fall into the *Low Improving* class for school experiences, suggesting a pattern in which females are more likely to have initially positive experiences but then either remain resilient or decline over time, whereas males were more likely to begin with lower functioning but improve during early adolescence. In the ABCD Study®, participants were assessed for ADHD diagnostic criteria but were not informed if they met criteria; therefore, these data reflect ADHD diagnostic thresholds while representing naturalistic patterns of access to diagnosis and treatment. Females with ADHD are frequently underrecognized during childhood and adolescence, in part because they are more likely to present with inattentive and internalizing symptom profiles rather than the more overt hyperactive and impulsive symptoms commonly observed in males [115]. However, while hyperactive symptoms decrease over time, inattentive symptoms often persist in both sexes [116,117]. These differing symptom profiles, along with “masking” or compensatory behaviors among females, can lead to delayed identification and treatment, despite comparable levels of impairment in cognitive and functional domains [117]. Furthermore, recent studies indicate that many females experience psychosocial challenges in adolescence but are not diagnosed with ADHD until adulthood, often after treatment for anxiety or depression [118].

These developmental patterns may help explain the trajectory differences observed in the present study. Females may initially appear to function well in childhood due to compensatory strategies or lower externalizing symptoms, but functioning may decline as environmental demands increase. Early adolescence represents a particularly important developmental period, as youth transition into school environments with greater academic demands, increased social complexity, and greater expectations for independence [115,119]. Prior research suggests that undiagnosed females with ADHD experience emerging difficulties during this stage, including declining self-esteem, increased internalizing symptoms, and challenges in peer and family relationships [50,52,120]. In contrast, males are more likely to be identified earlier due to disruptive symptom presentations, which may increase the likelihood of earlier diagnosis and treatment [115]. In addition, emerging evidence suggests that hormonal changes associated with puberty may exacerbate ADHD symptoms in some females, particularly emotional dysregulation and attentional difficulties, and differences in symptoms and cognitive functioning have been demonstrated in relation to the menstrual cycle, although these mechanisms remain poorly understood [119,121]. Overall, although females may demonstrate early strengths in some functional domains, they remain vulnerable to worsening family and school functioning across adolescence, particularly if symptoms are unrecognized and untreated [118,119]. These findings highlight the importance of improved screening and long-term follow-up for females with ADHD.

### Limitations

This study is strengthened by the large sample size, use of sample weights to improve generalizability, latent class analysis to identify latent population heterogeneity, and a novel examination of medication use patterns. However, several limitations should be noted. First, analyses were constrained by the measures available through the ABCD Study®, which were not designed around the present research. Second, medication use data are limited to the two-week period preceding each study time point and do not capture more granular details regarding dosage, day-to-day consistency of use, and intermittent patterns of use (e.g., medication “holidays” during summer break). Additionally, the ABCD Study® was only able to recruit participants located near study sites, therefore the results may not be generalizable to more rural populations [122]. Analytic requirements also necessitated the use of broad race and ethnicity categories, limiting interpretation given the heterogeneity within these groups. Future work should leverage more precise racial and ethnic data to better understand these findings. Finally, although centering child-reported outcomes addresses an important gap, cross-informant discrepancies are common in ADHD research and child-report may diverge from parent- or teacher-report [123]. Nevertheless, this study contributes valuable insight into the social and structural predictors of child-reported trajectories in a large, diverse, contemporary sample of children with ADHD.

## Conclusion

This study characterized observable patterns of medication use and latent trajectory classes of child-reported functional outcomes throughout early adolescence, and examined associations of sex, racial and ethnic identity, social determinants of health, and medication use with functional outcome trajectory classes. Findings indicate limited medication use in this cohort, with approximately half of participants never using ADHD medication across the six time points. Medication use varied in relation to demographic and SDOH factors; however, aside from associations that may reflect greater clinical complexity (i.e., nonstimulant medication use), medication use patterns were not significantly related to functional outcome trajectory classes as hypothesized. In contrast, perceived ethnic discrimination, racial and ethnic identity, sex, parental partnership and employment, and insurance status were significantly associated with functional outcome trajectories in children with ADHD, suggesting that risk and resilience factors linked to social and structural determinants of health may be particularly impactful for this population. Future research should further investigate the mechanisms underlying these associations and develop targeted, accessible, non-pharmacological interventions to reduce risk and promote resilience among children with ADHD who are vulnerable to disparate outcomes.

## Supporting information

S1 Table Demographic characteristics of participants retained vs lost to follow-up by T5. (DOCX)

S2 Table Medication use and associated demographic characteristics. (DOCX)

S3 Table LCGA Fit Indices. (DOCX)

S4 Table Latent Class Membership. (DOCX)

S5 Table Multinomial logistic regression results. (DOCX)
