# Supplementary material for: Medication use, social determinants of health, and latent trajectories of functional outcomes in children with attention-deficit/hyperactivity disorder
Source: PLoS One. 2026 Aug 5;21(8):e0350337. doi: 10.1371/journal.pone.0350337 (PMC13440877; doi:10.1371/journal.pone.0350337)
Supplement: Supplementary file 1 [file pone.0350337.s001.docx]

| **S1 Table: Demographic characteristics of participants retained vs lost to follow-up by T5** | | | | | | |
| --- | --- | --- | --- | --- | --- | --- |
| **Characteristic** | **Overall** N = 1,587*^1^* | **Lost to follow-up** N = 478*^1^* | | | **Retained at T5** N = 1,109*^1^* | **p-value***^2^* |
| **Sex** |  |  | | |  | >.900 |
| Male | 1063 (67%) | 321 (67%) | | | 742 (67%) |  |
| Female | 524 (33%) | 157 (33%) | | | 367 (33%) |  |
| **Racial and ethnic identity** | | | | | | .006 |
| White (non-Hispanic) | 291 (18%) | 90 (19%) | | | 201 (18%) |  |
| Hispanic | 853 (54%) | 229 (48%) | | | 624 (56%) |  |
| Black (non-Hispanic) | 237 (15%) | 90 (19%) | | | 147 (13%) |  |
| Another identity (non-Hispanic) | 206 (13%) | 69 (14%) | | | 137 (12%) |  |
| **Annual household income** | | | | | | <.001 |
| <$25,000 | 246 (16%) | 96 (20%) | | | 150 (14%) |  |
| $25,000-$50,000 | 237 (15%) | 84 (18%) | | | 153 (14%) |  |
| $50,000-$75,000 | 211 (13%) | 48 (10%) | | | 163 (15%) |  |
| $75,000-$100,000 | 219 (14%) | 63 (13%) | | | 156 (14%) |  |
| $100,000-$200,000 | 418 (26%) | 88 (18%) | | | 330 (30%) |  |
| >$200,000 | 130 (8.2%) | 43 (9.0%) | | | 87 (7.8%) |  |
| Don’t know | 66 (4.2%) | 34 (7.1%) | | | 32 (2.9%) |  |
| Prefer not to answer | 60 (3.8%) | 22 (4.6%) | | | 38 (3.4%) |  |
| **Highest parent education** |  | |  |  | | <.001 |
| Less than high school | 69 (4.3%) | 40 (8.4%) | | | 29 (2.6%) |  |
| High school | 130 (8.2%) | 42 (8.8%) | | | 88 (7.9%) |  |
| Vocational | 491 (31%) | 168 (35%) | | | 323 (29%) |  |
| Bachelor’s | 425 (27%) | 124 (26%) | | | 301 (27%) |  |
| Graduate or professional | 470 (30%) | 103 (22%) | | | 367 (33%) |  |
| Missing | 2 (0.1%) | 1 (0.2%) | | | 1 (<0.1%) |  |
| **Parental partnership and employment** | | | | | | <.001 |
| Partnered, 2 in labor force | 161 (10%) | 67 (14%) | | | 94 (8.5%) |  |
| Partnered, 1 in labor force | 313 (20%) | 108 (23%) | | | 205 (18%) |  |
| Partnered, 0 in labor force | 43 (2.7%) | 21 (4.4%) | | | 22 (2.0%) |  |
| Single, in labor force | 352 (22%) | 107 (22%) | | | 245 (22%) |  |
| Single, not in labor force | 693 (44%) | 167 (35%) | | | 526 (47%) |  |
| Missing | 25 (1.6%) | 8 (1.7%) | | | 17 (1.5%) |  |
| **ADI** | 41.79 (27.45) | 42.88 (27.87) | | | 41.32 (27.26) | .300 |
| Missing | 118 | 34 | | | 84 |  |
| ^1^ n (%); Mean (SD) | | | | | | |
| ^2^ Pearson’s Chi-squared test; independent two-sample t-test | | | | | | |
