# Supplementary material for: Medication use, social determinants of health, and latent trajectories of functional outcomes in children with attention-deficit/hyperactivity disorder
Source: PLoS One. 2026 Aug 5;21(8):e0350337. doi: 10.1371/journal.pone.0350337 (PMC13440877; doi:10.1371/journal.pone.0350337)
Supplement: Supplementary file 2 [file pone.0350337.s002.docx]

**S2 Table: Medication use and associated demographic characteristics**

|  | **Ever vs never used** | | | **Ever Users** | | | | | | | | | |
| --- | --- | --- | --- | --- | --- | --- | --- | --- | --- | --- | --- | --- | --- |
|  |  |  |  | **Age of initiation** | | | **Medication type** | | | **Consistency of use** | | | |
|  | **Never used**^1^ | **Ever used**^1^ | **p-value**^2^ | **Early**^1^ | **Late**^1^ | **p-value**^2^ | **Stimulant only**^1^ | **Some nonstimulant**^1^ | **p-value**^2^ | **Consistent**^1^ | **Inconsistent**^1^ | **Discontinued**^1^ | **p-value**^2^ |
|  | N = 796 | N = 789 |  | N = 518 | N = 271 |  | N= 485 | N = 304 |  | N = 196 | N = 188 | N = 405 |  |
| **Sex** | <.001 | | |  | .001 | |  | .278 | |  |  | .868 | |
| M | 501 (63) | 560 (71) |  | 388 (75) | 172 (63) |  | 337 (69) | 223 (73) |  | 137 (70) | 136 (72) | 287 (71) |  |
| F | 295 (37) | 229 (29) |  | 130 (25) | 99 (37) |  | 148 (31) | 81 (27) |  | 59 (30) | 52 (28) | 118 (29) |  |
| **Racial and ethnic identity** | | <.001 | |  | .048 | |  | .306 | |  |  | <.001 | |
| White | 383 (48) | 468 (59) |  | 322 (62) | 146 (54) |  | 279 (58) | 189 (62) |  | 149 (76) | 113 (60) | 206 (51) |  |
| Hispanic | 167 (21) | 124 (16) |  | 70 (14) | 54 (20) |  | 84 (17) | 40 (13) |  | 22 (11) | 29 (15) | 73 (18) |  |
| Black | 142 (18) | 95 (12) |  | 64 (12) | 31 (11) |  | 62 (13) | 33 (11) |  | 8 (4.1) | 22 (12) | 65 (16) |  |
| Another | 104 (13) | 102 (13) |  | 62 (12) | 40 (15) |  | 60 (12) | 42 (14) |  | 17 (8.7) | 24 (13) | 61 (15) |  |
| **Income** | | .007 | |  | .344 | |  | .060 | |  |  | <.001 | |
| <25 | 163 (20) | 126 (16) |  | 85 (16) | 41 (15) |  | 64 (13) | 62 (20) |  | 10 (5.1) | 32 (17) | 84 (21) |  |
| 25-50 | 145 (18) | 116 (15) |  | 86 (17) | 30 (11) |  | 69 (14) | 47 (15) |  | 19 (9.7) | 25 (13) | 72 (18) |  |
| 50-75 | 117 (15) | 104 (13) |  | 67 (13) | 37 (14) |  | 66 (14) | 38 (13) |  | 28 (14) | 25 (13) | 51 (13) |  |
| 75-100 | 106 (13) | 123 (16) |  | 79 (15) | 44 (16) |  | 78 (16) | 45 (15) |  | 38 (19) | 22 (12) | 63 (16) |  |
| 100-200 | 208 (26) | 241 (31) |  | 149 (29) | 92 (34) |  | 151 (31) | 90 (30) |  | 83 (42) | 62 (33) | 96 (24) |  |
| 200+ | 57 (7.2) | 79 (10) |  | 52 (10) | 27 (1.0) |  | 57 (12) | 22 (7.2) |  | 18 (9.2) | 22 (12) | 39 (9.6) |  |
| **Insurance** | | .167 | |  | .435 | |  | <.001 | |  |  | .020 | |
| Private | 441 (55) | 474 (60) |  | 311 (60) | 163 (60) |  | 311 (64) | 163 (54) |  | 139 (71) | 110 (59) | 225 (56) |  |
| Public | 256 (32) | 226 (29) |  | 145 (28) | 81 (30) |  | 114 (24) | 112 (37) |  | 41 (21) | 54 (29) | 131 (32) |  |
| Mult/other | 57 (7.2) | 59 (7.5) |  | 44 (8.5) | 15 (5.5) |  | 39 (8.0) | 20 (6.6) |  | 9 (4.6) | 18 (9.6) | 32 (7.9) |  |
| Uninsured | 42 (5.3) | 30 (3.8) |  | 18 (3.5) | 12 (4.4) |  | 21 (4.3) | 9 (3.0) |  | 7 (3.6) | 6 (3.2) | 17 (4.2) |  |
| **Education** | | <.001 | |  | .152 | |  | .040 | |  |  | <.001 | |
| < High school | 50 (6.3) | 20 (2.5) |  | 16 (3.1) | 4 (1.5) |  | 8 (1.6) | 12 (3.9) |  | 2 (1.0) | 4 (2.1) | 14 (3.5) |  |
| High school | 79 (9.9) | 50 (6.3) |  | 34 (6.6) | 16 (5.9) |  | 28 (5.8) | 22 (7.2) |  | 4 (2.0) | 9 (4.8) | 37 (9.1) |  |
| Vocational | 241 (30) | 249 (32) |  | 175 (34) | 74 (27) |  | 143 (29) | 106 (35) |  | 49 (25) | 69 (37) | 131 (32) |  |
| Bachelor’s | 209 (26) | 217 (28) |  | 137 (26) | 80 (30) |  | 147 (30) | 70 (23) |  | 51 (26) | 47 (25) | 119 (29) |  |
| Graduate, profess. | 217 (27) | 253 (32) |  | 156 (30) | 97 (36) |  | 159 (33) | 94 (31) |  | 90 (46) | 59 (31) | 104 (26) |  |
| **Parent partnership and employment^3^** | | .037 | |  | .495 | |  | <.001 | |  |  | <.001 | |
| 0 | 89 (11) | 76 (9.6) |  | 52 (10) | 24 (8.9) |  | 37 (7.6) | 39 (13) |  | 7 (3.6) | 22 (12) | 47 (12) |  |
| 1 | 170 (21) | 149 (19) |  | 101 (19) | 48 (18) |  | 97 (20) | 52 (17) |  | 20 (10) | 32 (17) | 97 (24) |  |
| 00 | 26 (3.3) | 19 (2.4) |  | 15 (2.9) | 4  (1.5) |  | 8  (1.6) | 11 (3.6) |  | 3 (1.5) | 3 (1.6) | 13 (3.2) |  |
| 01 | 189 (24) | 164 (21) |  | 110 (21) | 54 (20) |  | 85 (18) | 79 (26) |  | 30 (15) | 45 (24) | 89 (22) |  |
| 11 | 322 (40) | 381 (48) |  | 240 (46) | 141 (52) |  | 258 (53) | 123 (40) |  | 136 (69) | 86 (46) | 159 (39) |  |
| ^1^N (%)  ^2^Pearson's Chi-squared test (Fischer’s exact test was used when Chi-square assumptions were violated)  ^3^0 = single, not in the labor force; 1 = single, in the labor force; 00 = partnered, 0 partners in the labor force; 01 = partnered, 1 partner in the labor force; 11 = partnered, 2 partners in the labor force | | | | | | | | | | | | | |
