# Supplementary material for: Medication use, social determinants of health, and latent trajectories of functional outcomes in children with attention-deficit/hyperactivity disorder
Source: PLoS One. 2026 Aug 5;21(8):e0350337. doi: 10.1371/journal.pone.0350337 (PMC13440877; doi:10.1371/journal.pone.0350337)
Supplement: Supplementary file 3 [file pone.0350337.s003.docx]

**S3 Table: LCGA Fit Indices**

Notes: LL = log-likelihood. Lower AIC/BIC/CAIC/ssBIC generally indicate better fit. Higher E indicates clearer classification.

**Family conflict:**

| **# Classes (K)** | **N** | **# Params (p)** | **LogLik** | **Entropy** | **AIC** | **BIC** | **CAIC** | **ssBIC** | **CLC** | **ICL-BIC** | **NEC** | **E** |
| --- | --- | --- | --- | --- | --- | --- | --- | --- | --- | --- | --- | --- |
| 1 | 1,585 | 3 | -9,429.67 | 0.000 | 18,865.33 | 18,881.44 | 18,884.44 | 18,871.90 | 18,859.33 | 18,881.44 | 1.000 |  |
| 2 | 1,585 | 6 | -9,323.04 | 375.674 | 18,658.08 | 18,690.29 | 18,696.29 | 18,671.23 | 19,397.43 | 19,441.64 | 3.523 | 0.658 |
| 3 | 1,585 | 9 | -9,226.70 | 551.319 | 18,471.39 | 18,519.71 | 18,528.71 | 18,491.11 | 19,556.03 | 19,622.34 | 2.716 | 0.683 |
| 4 | 1,585 | 12 | -9,226.70 | 1,223.926 | 18,477.39 | 18,541.81 | 18,553.81 | 18,503.69 | 20,901.24 | 20,989.66 | 6.030 | 0.443 |
| 5 | 1,585 | 15 | -9,226.70 | 1,716.336 | 18,483.39 | 18,563.92 | 18,578.92 | 18,516.26 | 21,886.06 | 21,996.59 | 8.456 | 0.327 |
| 6 | 1,585 | 18 | -9,226.70 | 2,035.738 | 18,489.39 | 18,586.02 | 18,604.02 | 18,528.84 | 22,524.87 | 22,657.50 | 10.030 | 0.283 |

**Prosocial behavior:**

| **# Classes (K)** | **N** | **# Params (p)** | **LogLik** | **Entropy** | **AIC** | **BIC** | **CAIC** | **ssBIC** | **CLC** | **ICL-BIC** | **NEC** | **E** |
| --- | --- | --- | --- | --- | --- | --- | --- | --- | --- | --- | --- | --- |
| 1 | 1,584 | 3 | -9,569.57 | 0.000 | 19,145.15 | 19,161.25 | 19,164.25 | 19,151.72 | 19,139.15 | 19,161.25 | 1.000 |  |
| 2 | 1,584 | 6 | -9,489.86 | 377.911 | 18,991.73 | 19,023.93 | 19,029.93 | 19,004.87 | 19,735.55 | 19,779.76 | 4.741 | 0.656 |
| 3 | 1,584 | 9 | -9,389.81 | 550.298 | 18,797.61 | 18,845.92 | 18,854.92 | 18,817.33 | 19,880.21 | 19,946.52 | 3.061 | 0.684 |
| 4 | 1,584 | 12 | -9,389.81 | 1,210.062 | 18,803.61 | 18,868.02 | 18,880.02 | 18,829.90 | 21,199.73 | 21,288.15 | 6.731 | 0.449 |
| 5 | 1,584 | 15 | -9,389.81 | 1,737.394 | 18,809.61 | 18,890.13 | 18,905.13 | 18,842.47 | 22,254.40 | 22,364.91 | 9.665 | 0.318 |
| 6 | 1,584 | 18 | -9,389.81 | 2,049.334 | 18,815.61 | 18,912.23 | 18,930.23 | 18,855.05 | 22,878.28 | 23,010.90 | 11.400 | 0.278 |

**School experiences:**

| **# Classes (K)** | **N** | **# Params (p)** | **LogLik** | **Entropy** | **AIC** | **BIC** | **CAIC** | **ssBIC** | **CLC** | **ICL-BIC** | **NEC** | **E** |
| --- | --- | --- | --- | --- | --- | --- | --- | --- | --- | --- | --- | --- |
| 1 | 1,585 | 3 | -9,529.17 | 0.000 | 19,064.35 | 19,080.45 | 19,083.45 | 19,070.92 | 19,058.35 | 19,080.45 | 1.000 |  |
| 2 | 1,585 | 6 | -9,478.38 | 604.196 | 18,968.75 | 19,000.96 | 19,006.96 | 18,981.90 | 20,165.14 | 20,209.35 | 11.894 | 0.450 |
| 3 | 1,585 | 9 | -9,406.97 | 703.485 | 18,831.95 | 18,880.26 | 18,889.26 | 18,851.67 | 20,220.92 | 20,287.23 | 5.757 | 0.596 |
| 4 | 1,585 | 12 | -9,368.83 | 777.082 | 18,761.66 | 18,826.08 | 18,838.08 | 18,787.96 | 20,291.82 | 20,380.24 | 4.846 | 0.646 |
| 5 | 1,585 | 15 | -9,350.71 | 954.484 | 18,731.41 | 18,811.94 | 18,826.94 | 18,764.29 | 20,610.38 | 20,720.91 | 5.348 | 0.626 |
| 6 | 1,585 | 18 | -9,342.20 | 1,212.265 | 18,720.41 | 18,817.04 | 18,835.04 | 18,759.85 | 21,108.93 | 21,241.56 | 6.484 | 0.573 |
