# Supplementary material for: Medication use, social determinants of health, and latent trajectories of functional outcomes in children with attention-deficit/hyperactivity disorder
Source: PLoS One. 2026 Aug 5;21(8):e0350337. doi: 10.1371/journal.pone.0350337 (PMC13440877; doi:10.1371/journal.pone.0350337)
Supplement: Supplementary file 4 [file pone.0350337.s004.docx]

| **S4 Table: Latent Class Membership**  **Family Conflict** | | | | | |
| --- | --- | --- | --- | --- | --- |
|  | **Overall**  N = 1,585^1^ | **Resilient**  N = 1,136^1^ | **Declining** N = 204^1^ | **Low Improving** N = 245^1^ | **p-value**^2^ |
| **ADI** | 41.9 (26.82) | 40.7 (26.37) | 39.3 (24.82) | 50.1 (29.01) | <.001 |
| **Ethnic discrimination** | 1.2 (0.33) | 1.1 (0.29) | 1.2 (0.43) | 1.2 (0.40) | <.001 |
| **Racial and ethnic identity** | | | | | .096 |
| White (non-Hispanic) | 851 (54%) | 611 (54%) | 111 (54%) | 129 (53%) |  |
| Hispanic | 291 (18%) | 222 (20%) | 36 (18%) | 33 (13%) |  |
| Black (non-Hispanic) | 237 (15%) | 169 (15%) | 25 (12%) | 43 (18%) |  |
| Another identity (non-Hispanic) | 206 (13%) | 134 (12%) | 32 (16%) | 40 (16%) |  |
| **Income** |  |  |  |  | .016 |
| <$25,000 | 289 (18%) | 205 (18%) | 31 (15%) | 53 (22%) |  |
| $25,000-$50,000 | 261 (16%) | 166 (15%) | 39 (19%) | 56 (23%) |  |
| $50,000-$75,000 | 221 (14%) | 166 (15%) | 22 (11%) | 33 (13%) |  |
| $75,000-$100,000 | 229 (14%) | 161 (14%) | 32 (16%) | 36 (15%) |  |
| $100,000-$200,000 | 449 (28%) | 334 (29%) | 61 (30%) | 54 (22%) |  |
| >$200,000 | 136 (8.6%) | 104 (9.2%) | 19 (9.3%) | 13 (5.3%) |  |
| **Insurance status** |  |  |  |  | .123 |
| Private | 915 (58%) | 679 (60%) | 112 (55%) | 124 (51%) |  |
| Public | 482 (30%) | 331 (29%) | 63 (31%) | 88 (36%) |  |
| Uninsured or other | 72 (4.5%) | 49 (4.3%) | 13 (6.4%) | 10 (4.1%) |  |
| Multiple policies | 116 (7.3%) | 77 (6.8%) | 16 (7.8%) | 23 (9.4%) |  |
| **Parent education** |  |  |  |  | .005 |
| Less than high school | 70 (4.4%) | 53 (4.7%) | 8 (3.9%) | 9 (3.7%) |  |
| High school | 129 (8.1%) | 98 (8.6%) | 11 (5.4%) | 20 (8.2%) |  |
| Vocational or some college | 490 (31%) | 327 (29%) | 60 (29%) | 103 (42%) |  |
| Bachelor’s degree | 426 (27%) | 304 (27%) | 63 (31%) | 59 (24%) |  |
| Graduate degree | 470 (30%) | 354 (31%) | 62 (30%) | 54 (22%) |  |
| **Parent partnership and employment status** | | | | | .085 |
| Partnered, 2 in labor force | 703 (44%) | 522 (46%) | 94 (46%) | 87 (36%) |  |
| Partnered, 1 in labor force | 353 (22%) | 244 (21%) | 40 (20%) | 69 (28%) |  |
| Partnered, 0 in labor force | 45 (2.8%) | 29 (2.6%) | 6 (2.9%) | 10 (4.1%) |  |
| Single, in labor force | 319 (20%) | 220 (19%) | 47 (23%) | 52 (21%) |  |
| Single, not in labor force | 165 (10%) | 121 (11%) | 17 (8.3%) | 27 (11%) |  |
| **Sex** |  |  |  |  | .104 |
| Male | 1,061 (67%) | 766 (67%) | 124 (61%) | 171 (70%) |  |
| Female | 524 (33%) | 370 (33%) | 80 (39%) | 74 (30%) |  |
| **Age at initiation** |  |  |  |  | .295 |
| Early | 518 (33%) | 378 (33%) | 62 (30%) | 78 (32%) |  |
| Late | 271 (17%) | 192 (17%) | 44 (22%) | 35 (14%) |  |
| Never | 796 (50%) | 566 (50%) | 98 (48%) | 132 (54%) |  |
| **Medication type** |  |  |  |  | .201 |
| Stimulant only | 485 (31%) | 363 (32%) | 59 (29%) | 63 (26%) |  |
| Never used | 796 (50%) | 566 (50%) | 98 (48%) | 132 (54%) |  |
| Some nonstimulant use | 304 (19%) | 207 (18%) | 47 (23%) | 50 (20%) |  |
| **Consistency** |  |  |  |  | .475 |
| Consistent use | 196 (12%) | 146 (13%) | 29 (14%) | 21 (8.6%) |  |
| Never used | 796 (50%) | 566 (50%) | 98 (48%) | 132 (54%) |  |
| Discontinued | 405 (26%) | 294 (26%) | 49 (24%) | 62 (25%) |  |
| Inconsistent use | 188 (12%) | 130 (11%) | 28 (14%) | 30 (12%) |  |
| ^1^n (%); Mean (SD) | | | | | |
| ^2^Pearson's Chi-squared test; One-way ANOVA | | | | | |

| **Prosocial Behavior** | | | | | |
| --- | --- | --- | --- | --- | --- |
|  | **Overall**  N = 1,584^1^ | **Resilient**  N = 1,147^1^ | **Declining**  N = 260^1^ | **Low Improving**  N = 177^1^ | **p-value**^2^ |
| **ADI** | 41.9 (26.83) | 41.2 (26.74) | 46.6 (27.93) | 39.6 (25.07) | .009 |
| **Ethnic discrimination** | 1.2 (0.33) | 1.2 (0.30) | 1.2 (0.40) | 1.2 (0.40) | .146 |
| **Racial and ethnic identity** | | | | | .023 |
| White (non-Hispanic) | 850 (54%) | 624 (54%) | 134 (52%) | 92 (52%) |  |
| Hispanic | 291 (18%) | 197 (17%) | 55 (21%) | 39 (22%) |  |
| Black (non-Hispanic) | 237 (15%) | 185 (16%) | 38 (15%) | 14 (7.9%) |  |
| Another identity (non-Hispanic) | 206 (13%) | 141 (12%) | 33 (13%) | 32 (18%) |  |
| **Income** |  |  |  |  | .084 |
| <$25,000 | 289 (18%) | 192 (17%) | 67 (26%) | 30 (17%) |  |
| $25,000-$50,000 | 261 (16%) | 189 (16%) | 45 (17%) | 27 (15%) |  |
| $50,000-$75,000 | 220 (14%) | 167 (15%) | 28 (11%) | 25 (14%) |  |
| $75,000-$100,000 | 229 (14%) | 168 (15%) | 37 (14%) | 24 (14%) |  |
| $100,000-$200,000 | 449 (28%) | 334 (29%) | 65 (25%) | 50 (28%) |  |
| >$200,000 | 136 (8.6%) | 97 (8.5%) | 18 (6.9%) | 21 (12%) |  |
| **Insurance status** |  |  |  |  | .010 |
| Private | 914 (58%) | 680 (59%) | 125 (48%) | 109 (62%) |  |
| Public | 482 (30%) | 333 (29%) | 102 (39%) | 47 (27%) |  |
| Uninsured or other | 72 (4.5%) | 46 (4.0%) | 16 (6.2%) | 10 (5.6%) |  |
| Multiple policies | 116 (7.3%) | 88 (7.7%) | 17 (6.5%) | 11 (6.2%) |  |
| **Parent education** |  |  |  |  | .090 |
| Less than high school | 69 (4.4%) | 48 (4.2%) | 15 (5.8%) | 6 (3.4%) |  |
| High school | 129 (8.1%) | 97 (8.5%) | 25 (9.6%) | 7 (4.0%) |  |
| Vocational or some college | 490 (31%) | 341 (30%) | 83 (32%) | 66 (37%) |  |
| Bachelor’s degree | 426 (27%) | 319 (28%) | 69 (27%) | 38 (21%) |  |
| Graduate degree | 470 (30%) | 342 (30%) | 68 (26%) | 60 (34%) |  |
| **Parent partnership and employment status** | | | | | .687 |
| Partnered, 2 in labor force | 702 (44%) | 504 (44%) | 109 (42%) | 89 (50%) |  |
| Partnered, 1 in labor force | 353 (22%) | 253 (22%) | 64 (25%) | 36 (20%) |  |
| Partnered, 0 in labor force | 45 (2.8%) | 35 (3.1%) | 5 (1.9%) | 5 (2.8%) |  |
| Single, in labor force | 319 (20%) | 235 (20%) | 51 (20%) | 33 (19%) |  |
| Single, not in labor force | 165 (10%) | 120 (10%) | 31 (12%) | 14 (7.9%) |  |
| **Sex** |  |  |  |  | <.001 |
| Male | 1,061 (67%) | 730 (64%) | 191 (73%) | 140 (79%) |  |
| Female | 523 (33%) | 417 (36%) | 69 (27%) | 37 (21%) |  |
| **Age at initiation** |  |  |  |  | .025 |
| Early | 518 (33%) | 364 (32%) | 98 (38%) | 56 (32%) |  |
| Late | 271 (17%) | 187 (16%) | 41 (16%) | 43 (24%) |  |
| Never | 795 (50%) | 596 (52%) | 121 (47%) | 78 (44%) |  |
| **Medication type** |  |  |  |  | .004 |
| Stimulant only | 485 (31%) | 357 (31%) | 71 (27%) | 57 (32%) |  |
| Never used | 795 (50%) | 596 (52%) | 121 (47%) | 78 (44%) |  |
| Some nonstimulant use | 304 (19%) | 194 (17%) | 68 (26%) | 42 (24%) |  |
| **Consistency** |  |  |  |  | .320 |
| Consistent use | 196 (12%) | 138 (12%) | 35 (13%) | 23 (13%) |  |
| Never used | 795 (50%) | 596 (52%) | 121 (47%) | 78 (44%) |  |
| Discontinued | 405 (26%) | 281 (24%) | 75 (29%) | 49 (28%) |  |
| Inconsistent use | 188 (12%) | 132 (12%) | 29 (11%) | 27 (15%) |  |
| ^1^n (%); Mean (SD) | | | | | |
| ^2^Pearson's Chi-squared test; One-way ANOVA | | | | | |

| **School Experiences** | | | | | |
| --- | --- | --- | --- | --- | --- |
|  | **Overall**  N = 1,585^1^ | **Resilient**  N = 1,172^1^ | **Declining**  N = 230^1^ | **Low Improving**  N = 183^1^ | **p-value**^2^ |
| **ADI** | 41.9 (26.82) | 40.9 (26.84) | 46.0 (26.40) | 43.3 (26.85) | .025 |
| **Ethnic discrimination** | 1.2 (0.33) | 1.2 (0.32) | 1.2 (0.33) | 1.2 (0.41) | .179 |
| **Racial and ethnic identity** | | | | | .385 |
| White (non-Hispanic) | 851 (54%) | 635 (54%) | 119 (52%) | 97 (53%) |  |
| Hispanic | 291 (18%) | 199 (17%) | 51 (22%) | 41 (22%) |  |
| Black (non-Hispanic) | 237 (15%) | 182 (16%) | 31 (13%) | 24 (13%) |  |
| Another identity (non-Hispanic) | 206 (13%) | 156 (13%) | 29 (13%) | 21 (11%) |  |
| **Income** | | | | | .003 |
| <$25,000 | 289 (18%) | 204 (17%) | 55 (24%) | 30 (16%) |  |
| $25,000-$50,000 | 261 (16%) | 179 (15%) | 51 (22%) | 31 (17%) |  |
| $50,000-$75,000 | 221 (14%) | 164 (14%) | 28 (12%) | 29 (16%) |  |
| $75,000-$100,000 | 229 (14%) | 159 (14%) | 33 (14%) | 37 (20%) |  |
| $100,000-$200,000 | 449 (28%) | 355 (30%) | 50 (22%) | 44 (24%) |  |
| >$200,000 | 136 (8.6%) | 111 (9.5%) | 13 (5.7%) | 12 (6.6%) |  |
| **Insurance status** | | | | | <.001 |
| Private | 915 (58%) | 713 (61%) | 94 (41%) | 108 (59%) |  |
| Public | 482 (30%) | 327 (28%) | 101 (44%) | 54 (30%) |  |
| Uninsured or other | 72 (4.5%) | 51 (4.4%) | 10 (4.3%) | 11 (6.0%) |  |
| Multiple policies | 116 (7.3%) | 81 (6.9%) | 25 (11%) | 10 (5.5%) |  |
| **Parent education** | | | | | <.001 |
| Less than high school | 70 (4.4%) | 49 (4.2%) | 16 (7.0%) | 5 (2.7%) |  |
| High school | 129 (8.1%) | 89 (7.6%) | 26 (11%) | 14 (7.7%) |  |
| Vocational or some college | 490 (31%) | 343 (29%) | 77 (33%) | 70 (38%) |  |
| Bachelor’s degree | 426 (27%) | 308 (26%) | 64 (28%) | 54 (30%) |  |
| Graduate degree | 470 (30%) | 383 (33%) | 47 (20%) | 40 (22%) |  |
| **Parent partnership and employment status** | | | | | .454 |
| Partnered, 2 in labor force | 703 (44%) | 531 (45%) | 87 (38%) | 85 (46%) |  |
| Partnered, 1 in labor force | 353 (22%) | 266 (23%) | 49 (21%) | 38 (21%) |  |
| Partnered, 0 in labor force | 45 (2.8%) | 33 (2.8%) | 7 (3.0%) | 5 (2.7%) |  |
| Single, in labor force | 319 (20%) | 226 (19%) | 57 (25%) | 36 (20%) |  |
| Single, not in labor force | 165 (10%) | 116 (9.9%) | 30 (13%) | 19 (10%) |  |
| **Sex** | | | | | .006 |
| Male | 1,061 (67%) | 784 (67%) | 139 (60%) | 138 (75%) |  |
| Female | 524 (33%) | 388 (33%) | 91 (40%) | 45 (25%) |  |
| **Age at initiation** | | | | | .014 |
| Early | 518 (33%) | 380 (32%) | 75 (33%) | 63 (34%) |  |
| Late | 271 (17%) | 180 (15%) | 48 (21%) | 43 (23%) |  |
| Never | 796 (50%) | 612 (52%) | 107 (47%) | 77 (42%) |  |
| **Medication type** | | | | | .058 |
| Stimulant only | 485 (31%) | 350 (30%) | 74 (32%) | 61 (33%) |  |
| Never used | 796 (50%) | 612 (52%) | 107 (47%) | 77 (42%) |  |
| Some nonstimulant use | 304 (19%) | 210 (18%) | 49 (21%) | 45 (25%) |  |
| **Consistency** | | | | | .230 |
| Consistent use | 196 (12%) | 139 (12%) | 30 (13%) | 27 (15%) |  |
| Never used | 796 (50%) | 612 (52%) | 107 (47%) | 77 (42%) |  |
| Discontinued | 405 (26%) | 289 (25%) | 63 (27%) | 53 (29%) |  |
| Inconsistent use | 188 (12%) | 132 (11%) | 30 (13%) | 26 (14%) |  |
| ^1^n (%); Mean (SD) | | | | | |
| ^2^Pearson's Chi-squared test; One-way ANOVA | | | | | |
