# Supplementary material for: Medication use, social determinants of health, and latent trajectories of functional outcomes in children with attention-deficit/hyperactivity disorder
Source: PLoS One. 2026 Aug 5;21(8):e0350337. doi: 10.1371/journal.pone.0350337 (PMC13440877; doi:10.1371/journal.pone.0350337)
Supplement: Supplementary file 5 [file pone.0350337.s005.docx]

**S5 Table: Multinomial logistic regression results**

| **Variable** | **Family Conflict** | | **Prosocial Behavior** | | **School Experiences** | |
| --- | --- | --- | --- | --- | --- | --- |
|  | **Class 1 – *Declining***  **aOR (95% CI)** | **Class 2 – *Low* *improving***  **aOR (95% CI)** | **Class 1 – *Declining***  **aOR (95% CI)** | **Class 2 – *Low* *improving***  **aOR (95% CI)** | **Class 1 – *Declining***  **aOR (95% CI)** | **Class 2 – *Low improvin*g**  **aOR (95% CI)** |
| Notes: aOR = adjusted odds ratio. CI = confidence interval. * p < 0.05; ** p < 0.017 after Bonferroni correction | | | | | | |
| **(Intercept)** | 0.02  (0.00–0.10)** | 0.01  (0.00–0.04)** | 0.11  (0.03–0.44)* | 0.05  (0.01–0.25)** | 0.09  (0.02–0.36)** | 0.01  (0.00–0.05)** |
| **Demographic and SDOH Variables** | | | | | | |
| **ADI** | 0.99  (0.99–1.00) | 1.01  (1.00–1.02)** | 1.01  (1.00–1.01) | 1.00  (0.99–1.01) | 1.00  (1.00–1.01) | 1.00  (0.99–1.01) |
| **Ethnic discrimination** | 1.93  (1.29–2.88)** | 1.85  (1.27–2.70)** | 1.21  (0.82–1.80) | 1.60  (1.03–2.49)* | 1.26  (0.85–1.85) | 1.54  (1.00–2.37)* |
| **Racial and ethnic identity** | | | | | | |
| White (non-Hispanic) | Ref | Ref | Ref | Ref | Ref | Ref |
| Hispanic | 0.83  (0.54–1.27) | 0.55  (0.36–0.84)** | 1.12  (0.78–1.63) | 1.24  (0.81–1.88) | 1.06  (0.74–1.53) | 1.17  (0.77–1.77) |
| Black (non-Hispanic) | 0.75  (0.42–1.33) | 0.75  (0.47–1.19) | 0.74  (0.46–1.18) | 0.38  (0.19–0.76)** | 0.45  (0.27–0.74)** | 0.61  (0.34–1.11) |
| Another identity (non-Hispanic) | 0.86  (0.48–1.54) | 0.90  (0.54–1.49) | 1.04  (0.61–1.76) | 1.27  (0.72–2.22) | 0.50  (0.28–0.91)* | 0.63  (0.32–1.23) |
| **Income** | 0.96  (0.81–1.12) | 0.96  (0.83–1.10) | 0.95  (0.83–1.10) | 0.96  (0.82–1.13) | 0.97  (0.84–1.12) | 0.98  (0.83–1.16) |
| **Insurance status** | | | | | | |
| Private | Ref | Ref | Ref | Ref | Ref | Ref |
| Public | 1.30  (0.84–2.02) | 1.03  (0.69–1.53) | 1.57  (1.07–2.31)* | 0.94  (0.59–1.49) | 2.44  (1.64–3.62)** | 0.89  (0.57–1.40) |
| Multiple policies | 1.37  (0.72–2.60) | 1.47  (0.86–2.51) | 1.17  (0.66–2.07) | 0.97  (0.50–1.89) | 2.69  (1.60–4.51)** | 0.47  (0.21–1.08) |
| Uninsured or other | 1.07  (0.49–2.31) | 0.80  (0.38–1.71) | 1.58  (0.83–3.02) | 1.37  (0.65–2.89) | 1.11  (0.51–2.41) | 1.25  (0.58–2.68) |
| **Parent education** | 1.08  (0.90–1.31) | 1.12  (0.94–1.32) | 1.04  (0.88–1.22) | 1.12  (0.93–1.36) | 0.89  (0.75–1.04) | 0.93  (0.77–1.13) |
| **Parent partnership and employment status** | | | | | | |
| Partnered, 2 in labor force | Ref | Ref | Ref | Ref | Ref | Ref |
| Partnered, 1 in labor force | 0.83  (0.53–1.29) | 1.21  (0.81–1.79) | 1.09  (0.74–1.58) | 0.83  (0.53–1.30) | 1.00  (0.66–1.51) | 0.88  (0.56–1.39) |
| Partnered, 0 in labor force | 0.81  (0.31–2.08) | 1.24  (0.60–2.56) | 0.33  (0.13–0.85)* | 0.97  (0.42–2.25) | 0.65  (0.28–1.51) | 1.33  (0.57–3.09) |
| Single, in labor force | 1.03  (0.66–1.62) | 0.93  (0.60–1.43) | 0.68  (0.44–1.03) | 0.87  (0.54–1.40) | 1.29  (0.85–1.94) | 0.88  (0.53–1.43) |
| Single, not in labor force | 0.58  (0.31–1.07) | 0.71  (0.42–1.22) | 0.64  (0.38–1.06) | 0.80  (0.43–1.48) | 0.81  (0.49–1.35) | 0.81  (0.44–1.48) |
| **Sex** | | | | | | |
| Male | Ref | Ref | Ref | Ref | Ref | Ref |
| Female | 1.50  (1.08–2.07)** | 0.91  (0.66–1.24) | 0.58  (0.42–0.79)** | 0.47  (0.32–0.70)** | 1.36  (1.01–1.83)* | 0.65  (0.45–0.95)* |
| **Medication characteristics** | | | | | | |
| **Never used** | 1.12  (0.91–1.37) | 1.16  (0.94–1.42) | 0.95  (0.79–1.13) | 1.00  (0.81–1.23) | 0.90  (0.75–1.07) | 0.88  (0.72–1.08) |
| **Age at initiation** | | | | | | |
| Early | Ref | Ref | Ref | Ref | Ref | Ref |
| Late | 1.31  (0.82–2.11) | 0.85  (0.53–1.36) | 0.83  (0.54–1.28) | 1.31  (0.81–2.11) | 1.22  (0.79–1.87) | 1.53  (0.96–2.43) |
| **Medication type** | | | | | | |
| Stimulant only | Ref | Ref | Ref | Ref | Ref | Ref |
| Some nonstimulant use | 1.77  (1.11–2.81)** | 1.20  (0.78–1.84) | 1.72  (1.15–2.57)** | 1.57  (0.99–2.49) | 1.11  (0.74–1.68) | 1.09  (0.70–1.70) |
| **Consistency** | | | | | | |
| Consistent use | Ref | Ref | Ref | Ref | Ref | Ref |
| Inconsistent use | 1.05  (0.55–1.99) | 1.76  (0.93–3.32) | 0.79  (0.44–1.42) | 1.16  (0.61–2.20) | 0.91  (0.51–1.62) | 1.09  (0.58–2.04) |
| Discontinued | 0.79  (0.45–1.38) | 1.24  (0.70–2.21) | 0.92  (0.56–1.50) | 0.88  (0.50–1.55) | 0.71  (0.43–1.18) | 0.80  (0.46–1.39) |
| **Clinical characteristics** | | | | | | |
| **Hyperactive symptoms** | | | | | | |
| Intercept | 0.93  (0.79–1.11) | 1.02  (0.87–1.20) | 0.83  (0.72–0.97)* | 1.00  (0.84–1.20) | 0.77  (0.65–0.90)** | 0.96  (0.80–1.14) |
| Slope | 0.75  (0.21–2.70) | 1.63  (0.49–5.39) | 0.62  (0.19–1.97) | 3.22  (0.86–12.06) | 0.15  (0.04–0.50)** | 0.22  (0.06–0.82)* |
| Quadratic | 0.64  (0.53–0.76)** | 0.58  (0.49–0.69)** | 0.77  (0.66–0.91)** | 0.71  (0.59–0.85)** | 0.76  (0.64–0.89)** | 0.58  (0.48–0.70)** |
| **Inattentive symptoms** | | | | | | |
| Intercept | 1.04  (0.91–1.20) | 0.95  (0.83–1.09) | 1.12  (0.99–1.26)* | 0.95  (0.83–1.10) | 1.23  (1.09–1.39)** | 1.01  (0.87–1.16) |
| Slope | 2.02  (0.73–5.64) | 0.69  (0.26–1.84) | 0.67  (0.26–1.73) | 0.19  (0.06–0.58)** | 4.45  (1.74–11.36)** | 1.52  (0.51–4.52) |
| Quadratic | 0.56  (0.44–0.70)** | 0.50  (0.40–0.62)** | 0.72  (0.58–0.88)** | 0.63  (0.50–0.81)** | 0.69  (0.56–0.85)** | 0.49  (0.39–0.63)** |
| **Baseline comorbid symptoms** | | | | | | |
| **Internalizing** | 0.99  (0.98–1.01) | 0.99  (0.97–1.01) | 0.99  (0.97–1.00) | 0.99  (0.97–1.01) | 1.00  (0.99–1.02) | 1.04  (1.02–1.06)** |
| **Externalizing** | 1.03  (1.01–1.05)** | 1.04  (1.03–1.06)** | 1.02  (1.00–1.04)** | 1.02  (1.00–1.05)* | 1.01  (0.99–1.02) | 1.01  (0.99–1.03) |
